# Dose Mapping and Accumulation for Re-Irradiation of Lung Cancers: Methodological Principles, Uncertainties, Quality Assurance, and Practical Clinical Considerations

**DOI:** 10.3390/jcm15197527

**Published:** 2026-09-28

**Authors:** Mihaela Rosu-Bubulac, Hualiang Zhong, Indrin Julian Chetty

**Affiliations:** 1 Department of Radiation Oncology, Virginia Commonwealth University, 1001 East Leigh Street Rm 3-089, Richmond, VA 23219, USA; mihaela.rosu@vcuhealth.org; 2 Department of Radiation Oncology, Medical College of Wisconsin, 8701 W Watertown Plank Rd., Milwaukee, WI 53226, USA; hzhong@mcw.edu; 3 Department of Radiation Oncology, Cedars-Sinai Medical Center, 8700 Beverly Boulevard North Tower Lower Level, Los Angeles, CA 90048, USA

**Keywords:** lung cancer, thoracic re-irradiation, dose accumulation, dose mapping, deformable image registration, EQD2, BED, adaptive radiotherapy, quality assurance

## Abstract

**Background/Objectives:** Thoracic re-irradiation is increasingly considered for recurrent, persistent, or new lung cancers after prior radiation therapy. The central clinical-technical challenge is relating prior and prospective doses across changes in anatomy, breathing state, fractionation, and imaging. **Methods:** This clinical-technical Perspective synthesizes consensus guidance, technical and the methodological literature, and representative clinical evidence to discuss rigid and deformable registration, dose mapping, biological normalization with EQD2/BED, uncertainty, quality assurance, and practical decision-making in lung re-irradiation. **Results:** Cumulative dose is an estimate with inherent uncertainty whose clinical value depends on the clinical question, anatomic stability, tissue correspondence, dose-transfer method, biological assumptions, and the consequence of error. Published cumulative dose constraints can provide useful orientation, but they are not universal pass/fail thresholds and should be interpreted together with their original endpoints, assumptions, and accumulation method. **Conclusions:** A focused, risk-adapted workflow can support more defensible use of cumulative dose in lung re-irradiation. Practical considerations in this article are intended to complement formal consensus guidance, institutional protocols, or patient-specific multidisciplinary judgment.

## 1. Introduction: Why Cumulative Dose Is the Central Problem in Thoracic Re-Irradiation

Thoracic re-irradiation is one of the more difficult problems in radiation oncology [1,2,3,4,5]. It is often considered when another local treatment is clinically indicated, but the surrounding normal tissues have already received a substantial radiation burden. Re-irradiation is considered in several clinical scenarios: early-stage non-small cell lung cancer (NSCLC) recurring after prior SBRT; a new primary tumor arising near a previously treated region; locoregional recurrence after definitive chemoradiation; oligometastatic disease near old thoracic fields; or persistent disease after an incomplete response to treatment. Across these scenarios, the central challenge is estimating the prior and planned doses to nearby OARs with enough accuracy to determine whether another course of radiation can be delivered safely, without exceeding cumulative OAR dose constraints.

The field has increasingly recognized the need for shared terminology and a more consistent workflow. The ESTRO-EORTC consensus defines re-irradiation broadly as a new course of radiotherapy to a previously irradiated volume, or as a new course in which cumulative dose creates a toxicity concern [6]. That definition is helpful because it keeps both problems in view: spatial overlap and cumulative toxicity. Recent ESTRO technical guidance on cumulative dose evaluation makes the same point from a technical perspective: safe re-irradiation requires an appropriate choice among three-dimensional dose summation, conservative alternatives, radiobiologic rescaling, documentation, and uncertainty review [7]. The re-irradiation collaborative group (ReCOG) consensus guidance has also moved the field toward clearer standards for evaluating and reporting dose across multiple radiation courses [8].

Cumulative dose assessment in the thorax is difficult because each organ at risk has a different dose tolerance pattern. For the lung, the concern is not only the total irradiated volume, but also how much functional reserve remains after tumor, emphysema, surgery, atelectasis, prior radiation, pneumonitis, or fibrosis. For more serial structures, such as the spinal cord, proximal bronchial tree, esophagus, great vessels, and portions of the heart, even a small region of high cumulative dose may be clinically important. The chest wall and ribs are of concern in peripheral SBRT, where steep dose gradients can place a small volume at high risk. Therefore, the main planning issue is related to the location of the prior and prospective high-dose overlap regions and the level of confidence in the cumulative OAR dose estimates [9].

Modern treatment planning systems have tools to facilitate importing of images, their registration to a new planning CT, transfer of prior dose, biological dose conversion, and display of cumulative dose-volume histograms (DVH’s) [10]. The resulting doses in this workflow depend on assumptions that cannot always be easily verified. Uncertainties can be multi-factorial; some are related to spatial variability, such as the degree of alignment between prior and current image anatomy, the respiratory phase used, the accuracy of the deformable registration algorithm (DIR) in the face of sliding interfaces and density and mass changes, and the location of small high-dose regions. Other uncertainties can be linked to dosimetric or biologic variability, e.g., dose-grid resolution, interpolation methods, alpha-beta ratio, fractionation corrections, and assumptions about the time required for normal tissues to recover after the initial treatment (tissue recovery factors). Additional uncertainties can relate to treatment history, including the degree of agreement between planned and delivered prior doses, whether all prior dose fractions were delivered as intended, and the level of information pertaining to the prior treatment available to the clinical team for the re-treatment. Multi-institutional data show substantial variation in cumulative dose assessment across centers, reinforcing that methodology can affect cumulative dose interpretation and, in some cases, clinical decisions [11].

This Perspective approaches dose mapping and accumulation in lung re-irradiation as a clinical-technical process that depends on the quality of each step in the workflow. DIR is one part of that process, but its usefulness depends on the clinical setting, the available imaging, the amount of anatomic change, the organs at risk involved, the immobilization technique used, and the clinical decision tradeoffs. In some cases, accumulated dose can reasonably guide plan evaluation and replanning. In others, it should be treated only as a qualitative or semi-quantitative aid. When anatomy has changed substantially, when serial organs are close to high-dose overlap regions, or when the registration is difficult to validate, a conservative point-dose estimate or scenario-based judgment may be more appropriate than a single deformably accumulated estimate [7,8]. In practical terms, the dose accumulation method should fit the case: the higher the risk, and the more the result may affect the plan, the more carefully the accumulated dose needs to be verified. Figure 1 lays out the chain of assumptions and the points where uncertainty affects the cumulative-dose estimate.

## 2. Framing the Cumulative Dose Problem

This paper focuses on dose mapping and accumulation for lung cancer re-irradiation in early-stage, locally advanced, recurrent, and metastatic settings. The main emphasis is photon-based external beam radiotherapy, including SBRT, IMRT, VMAT, and adaptive radiotherapy [12,13,14,15]. Proton re-irradiation and PET-guided re-treatment are included as future directions and as examples of settings in which cumulative dose assessment becomes more complicated by physical dose, relative biological effectiveness, range uncertainty, and functional imaging. Formal consensus or guideline recommendations are identified by citation; otherwise, workflow suggestions are presented as practical considerations based on the available technical and clinical literature.

The re-irradiation problem varies by clinical setting, as summarized in Table 1. In each case, the first step is to define which prior dose information is relevant to the new treatment. This includes reviewing prior radiation courses, imaging, structures, dose files, and delivery records, and assessing the reliability of that information. The review may also include distance assessments between the recurrent targets and prior treatment sites to, in some instances, avoid exercising unnecessary detailed procedures for estimating the accumulated dose.

After the relevant prior data are identified, the next step is to decide how the prior and current treatments should be combined [16]. Depending on the case, this may involve rigid registration, deformable registration, point-dose summation, field-overlap review, or a hybrid approach. When fractionation differs between courses and a biologically meaningful cumulative comparison is needed, a common practical approach is to express dose on a shared biological scale, such as EQD2 or BED, before cumulative dose is interpreted. Physical-dose or qualitative field-overlap review may still answer selected spatial questions. This step is essential for identifying cumulative dose metrics that are sufficiently reliable to inform safe treatment planning, while clearly delineating areas where uncertainty remains. Such uncertainties should weigh into clinical decision-making. Re-irradiation decisions depend on dose, but also on disease status, treatment intent, pulmonary reserve, time since prior radiation, prior toxicity, performance status, and patient goals. For this reason, cumulative dose estimates should be presented with the method and assumptions used to generate them. Table 1 summarizes common lung re-irradiation scenarios and the cumulative-dose question most relevant to each.

The 2025 American Radium Society (ARS) Appropriate Use Criteria provide a contemporary synthesis of proposed composite EQD2 constraints for thoracic re-irradiation and can serve as a practical reference for organ-specific dose considerations [5]. Recommended composite constraints include esophagus V60 < 40% and Dmax < 100 Gy, lung V20 < 40%, heart V40 < 50% with mean dose kept as low as reasonably achievable, aorta/great vessels Dmax < 120 Gy, trachea/proximal bronchial tree Dmax < 110 Gy, spinal cord Dmax < 57 Gy, and brachial plexus Dmax < 85 Gy [5]. These recommendations are based on limited and heterogeneous clinical evidence and therefore remain context-dependent rather than definitive cumulative tolerance thresholds. Their interpretation depends on the reported dose endpoint, fractionation, spatial overlap between treatment courses, dose-accumulation method, and associated uncertainty. The ARS notes that these composite constraints were derived largely from conventionally fractionated thoracic re-irradiation experience, emphasizes individualized clinical assessment, and acknowledges the limited prospective evidence underlying the recommendations. Only 3 of the 52 studies included in the ARS review were prospective, and the authors note that further prospective studies are needed to strengthen these recommendations [5].

## 3. Methodological Foundations of Image Registration

Dose accumulation begins with image registration. Registration defines how anatomy from a previous treatment course is related to anatomy in the current course.

Rigid image registration (RIR) aligns two datasets using translations and rotations. It preserves distances and assumes that the relevant anatomy behaves as a single rigid body. In thoracic re-irradiation, rigid registration may be adequate when the region of interest is small, when the prior and current scans were acquired in similar positions and respiratory states, and when the relevant OARs have not changed substantially in shape or location. A small peripheral recurrence beside a prior peripheral field may be reasonably assessed with rigid registration if lung inflation, rib and chest-wall anatomy, and the target region are stable. Rigid registration is transparent, reproducible, and easy to review.

The limits of RIR become clear when different parts of the thorax move differently. The lung deforms with respiration. The mediastinum may shift with atelectasis or pleural effusion. The esophagus can change position and shape. The bronchial tree may remain stable relative to the central airways while moving differently relative to a peripheral target. The appearance and localization of cardiac structures may be altered by implanted cardiac devices and postsurgical changes. A match optimized to the target may misrepresent the spinal cord or esophagus; a match optimized to the spine may misrepresent the lesion. Organ-specific rigid registrations may therefore be preferable to a single global match, particularly when serial OAR maximum-dose estimates drive the decision [7].

Deformable image registration (DIR) allows various parts of the anatomy to move by different amounts. This can be useful in lung re-irradiation because the tumor, lung, mediastinum, diaphragm, and chest wall may not have the same shape or position as they did during the prior treatment. When the registration is reliable, the prior dose can be mapped onto the current planning CT for developing the new plan.

DIR is a model-based estimate of voxel correspondence between two image datasets, performed through a transformation referred to as a displacement or deformation vector field (DVF). In many thoracic regions, that correspondence is hard to define. A voxel of lung parenchyma on one CT may represent a changing mixture of air, blood, tissue, fibrosis, inflammation, or tumor. An atelectatic lung may re-expand. Tumor/s may shrink and be replaced by aerated lung, fibrosis, necrosis, or scar. Pleural fluid may appear or resolve. Airways may open or collapse. In these situations, the anatomy has not simply shifted or stretched; the underlying tissue may have changed in composition, density, or mass. This is important because dose is deposited in tissue, and a CT image does not always represent the same tissue from one scan to another. If anatomy has changed through tissue loss, tissue gain, aeration, collapse, or replacement, propagating dose values through a DVF may not preserve the physical or biological meaning of the original dose distribution [17,18,19,20,21,22]. A DVF can look acceptable on routine review yet still give misleading cumulative dose estimates in clinically important regions. Good image alignment does not, by itself, prove that the mapped dose is reliable [20,23].

Figure 2 is a synthetic conceptual illustration depicting categories of anatomic change relevant to lung re-irradiation. It highlights examples such as atelectasis, fibrosis, surgery, pleural fluid, and tumor response to illustrate how evolving thoracic anatomy can complicate image registration and cumulative dose mapping.

## 4. DIR Algorithm Classes and Lung-Specific Tradeoffs

DIR methods differ in the assumptions they make, and those assumptions should be understood before choosing a registration strategy for lung re-irradiation. Intensity-based algorithms align images by maximizing similarity between voxel intensity patterns, using metrics such as squared differences, cross-correlation, or mutual information. Thoracic CT-to-CT registration benefits from the strong contrast among air, soft tissue, bone, and tumor. The concern is that a change in CT intensity does not always mean that tissue has moved; it may mean that the tissue itself has changed. Tumor response, atelectasis resolution, pneumonitis, fibrosis, pleural effusion, variable contrast, and variable lung inflation can change Hounsfield units without representing true displacement. If an algorithm is pushed to align collapsed lung with aerated lung, it may generate a smooth but nonphysical transformation [24,25,26,27,28,29,30,31].

B-spline and free-form deformation methods describe deformation with a grid of control points. They are widely used because they can represent smooth anatomic change and can be regularized to reduce unrealistic warping. Their behavior depends on control-point spacing, regularization strength, optimization criteria, and DVF constraints. In lung applications, a coarse grid may miss local deformation near a tumor, bronchus, or chest wall. If the grid is fine (of higher spatial resolution), the registration has more freedom to match small details on the images. That can be useful, but it can also make the algorithm follow noise, partial-volume effects, or density changes that do not represent true tissue motion. Too much smoothing may also flatten sliding motion at pleural interfaces, where neighboring tissues move differently.

Demons-based and optical-flow-like methods estimate local motion from changes in image intensity. They can work well when images exhibit high contrast, the same anatomy is present on both scans and has mainly shifted or deformed. The concern is that intensity differences in the thorax do not always mean motion. They may reflect tumor regression, re-expansion of atelectatic lung, changing tumor borders, air-tissue interfaces, or post-radiation inflammation. In those settings, the calculated displacement may look reasonable but may not represent the same tissue on the two scans.

Contour- and feature-guided methods use added information, such as landmarks, organ surfaces, or clinician-drawn structures, to help guide the registration. In the thorax, this can be useful when the anatomy of interest is difficult to align from image intensity alone. Examples include the spinal canal, proximal bronchial tree, esophagus, heart, great vessels, ribs, chest wall, and target region.

These methods can help make the registration more clinically focused, especially when the main question involves a specific organ or high-dose region. However, good contour agreement does not mean that dose has been mapped correctly at every voxel. For example, matching the esophageal outline does not guarantee that the same part of the esophageal wall has been matched. Similarly, matching the bronchial tree does not guarantee accurate dose mapping to a small airway segment near a steep SBRT dose gradient. Contour guidance is therefore useful for registration review, but it does not by itself confirm that the accumulated dose is accurate.

Biomechanical models use physical rules to describe how tissues move, including elasticity, boundary conditions, organ motion, and sliding between surfaces. They are relevant in the thorax because breathing and pleural motion do not always behave well in purely intensity-based registration. In some cases, adding mechanical constraints may give a more realistic description of lung and chest motion. However, these models still rely on assumptions about tissue behavior, and those assumptions may be less reliable when anatomy has been changed by tumor, fibrosis, emphysema, surgery, pleural disease, or prior radiation.

Hybrid methods combine intensity information, contours, landmarks, biomechanical constraints, or regularization. In lung re-irradiation, this type of complementary information can be useful toward more accurate registration in complex anatomy. A registration may need to align bone for spinal cord review, airways for central bronchial structures, the target region for high-dose overlap, and the lung surface for pleural or chest-wall questions. The tradeoff is complexity; hybrid workflows may be harder to commission, validate, and document, and they may increase variation between users. The main question is whether the added complexity improves the cumulative dose metric used for treatment planning. Table 2 summarizes the main DIR algorithm classes and the tradeoffs most relevant to lung re-irradiation.

## 5. Dose Mapping Strategies: What Is Being Transferred, Preserved, or Assumed?

After registration, the next question is what should actually be transferred. Direct dose mapping moves dose values from the prior dose grid onto the current image, using the registration and interpolation. This is relatively simple and is available in many commercial systems. Clinically, it can help show where the old high-dose region sits relative to the new anatomy. The limitation is that dose is not simply attached to an image voxel. It represents energy deposited in tissue during a specific treatment course and in a specific anatomy. When anatomy has shifted or deformed without major density or tissue change, direct dose mapping may be a reasonable approximation. When tissue has been lost, gained, aerated, collapsed, or replaced, direct dose interpolation may no longer preserve the physical meaning of the original dose [10,17,18,19,20,21,22,26].

Voxel warping uses the DVF to place the prior dose onto the current anatomy. This can be useful because it shows where the old and new dose regions overlap and allows a cumulative DVH to be generated [32,33]. The main limitation is that the result is only as good as the deformation used to move the dose. If the registration is slightly wrong, especially near a steep SBRT dose gradient, the cumulative dose may be misleading. This matters most near structures where small high-dose regions can be clinically important, such as the central airways, esophagus, great vessels, spinal cord, and chest wall [27,28,29,30,31,34].

Mass- and energy-based mapping is based on the physical definition of dose: energy deposited per unit mass. These methods try to handle dose transfer more carefully than simple interpolation, especially when tissue density or volume has changed [17,18,19]. This is relevant in the lung because density changes are common: a collapsed lung may re-expand, a tumor may cavitate, fibrosis may contract tissue, and pleural fluid may resolve. However, these methods are still not widely used in routine clinical practice, and they still depend on assumptions about which tissue on one scan corresponds to tissue on another. They also cannot solve every biological problem created by changing anatomy. For example, if a previously treated tumor region later appears as aerated lung or scar, a true voxel-to-voxel match may not exist. Interpreting the cumulative dose then requires clinical and biological assumptions about what tissue is present and how prior dose should be counted [35,36]. These approaches can improve physical consistency relative to simple dose interpolation in selected settings, but they do not constitute a ground-truth solution. Their validity still depends on image quality, density estimation, transformation behavior, implementation details, and—most importantly—whether meaningful tissue correspondence exists between scans. They address one part of the physical dose-transfer problem and require independent commissioning and patient-specific validation.

Biological dose conversion is important when treatment courses use different fraction sizes, as often occurs when prior conventional radiation is followed by SBRT, hypofractionation, or another course of conventional treatment [6,7,8,16,37]. The same physical dose can have different biological effects depending on dose per fraction, alpha-beta ratio, repair, repopulation, and time between courses. For biologically meaningful cumulative OAR assessment across different fractionation schedules, doses should be converted to a common biological scale, such as EQD2 or BED, before they are added or compared. For metric-based cumulative mean EQD2 assessment, each course’s physical dose distribution is first converted voxel-wise to EQD2 using its fractionation and the selected organ-specific α/β ratio. Mean EQD2 is then calculated over the relevant OAR using consistent contour definitions, and the course-specific mean EQD2 values are added. EQD2 and BED are therefore best regarded as normalization tools that facilitate comparison across fractionation schedules; they do not establish that two spatially mapped dose distributions are biologically equivalent or that a cumulative tolerance has been clinically validated.

The calculation is simple in concept, but the choices behind it are not. Different organs may require different alpha-beta values; values used for lung, esophagus, spinal cord, heart, bronchial tree, chest wall, and great vessels should not be assumed to be interchangeable. Voxel-by-voxel conversion can also create artificial jumps at structure borders when different alpha-beta values are applied to neighboring tissues. Assumptions about tissue recovery add another source of uncertainty and are not well established for many thoracic organs, outside better-studied settings such as the central nervous system and spinal cord [38]. A complete report should state the model used, the alpha-beta values, the dose and fractionation for each course, whether recovery was assumed, how it was applied, and whether cumulative constraints were evaluated in EQD2, BED, or physical dose. Table 3 summarizes the main dose-mapping strategies and their best uses and limitations. Additional caution is warranted for very large doses per fraction, where extrapolation of the linear-quadratic model is less certain, and when a whole-organ average is compared with a small-volume or serial-organ endpoint. Organ-specific alpha/beta values are themselves estimates, and voxel-wise EQD2/BED conversion is a numerical transformation rather than validation of voxel-wise biological equivalence or tissue correspondence.

Alternative radiobiological formalisms have been proposed for very large doses per fraction, including the linear-quadratic-linear (LQ-L) model and the universal survival curve (USC), both of which modify the high-dose behavior of the conventional LQ model [39,40]. Their potential relevance is greatest for stereotactic treatments in which dose per fraction is well beyond conventional fractionation. However, clinical analyses have not established a universal replacement for the LQ model, and some large SBRT datasets remain consistent with LQ-based predictions [41]. More importantly for re-irradiation, alternative models do not yet have broadly validated organ-specific cumulative tolerance endpoints or recovery functions. Accordingly, they may be useful for sensitivity analyses or research comparisons when high-dose extrapolation materially affects the conclusion, but they should not be treated as interchangeable with EQD2/BED-based cumulative constraints.

Time-dependent recovery is best treated as a separate biological assumption rather than implicitly incorporated into the cumulative dose. The spinal cord is the best-studied example, for which a commonly used model assumes approximately 25% recovery after 6 months and 50% after 12 months; these values are based on preclinical and retrospective clinical evidence rather than a universally validated recovery function [38]. Comparable quantitative recovery models are not well established for thoracic OARs. Recovery is influenced by treatment interval, initial dose, and fractionation, and each treatment course therefore requires biological dose conversion before cumulative assessment. When a recovery factor is applied, the assumed model, interval, dose-fractionation basis, and effect on the cumulative result are important to report. When no validated organ-specific recovery model is available, an unadjusted (no-recovery) cumulative value can provide a transparent reference; if recovery could materially change the interpretation, comparison with one or more plausible recovery assumptions can be reported as a sensitivity analysis.

## 6. Lung-Specific Challenges in Cumulative Dose Assessment

Lung re-irradiation is strongly affected by changes in anatomy and breathing. Prior and current planning images may represent different respiratory states, including free breathing, 4DCT phases, average or maximum intensity projections, breath hold, gated imaging, or CBCT. When those states do not match, registration may align anatomy that was not actually in the same physical condition during treatment. This can affect target location, OAR position, and dose calculation and accumulation. In SBRT, where dose gradients are steep and margins are small, even modest respiratory mismatch can meaningfully change the cumulative dose estimate [33,42].

Tumor and lung changes create another problem. A prior tumor may shrink, cavitate, scar, progress, or disappear. Atelectasis may resolve, pleural fluid may clear, airways may open, and the lung may re-expand. In these situations, the key question is what tissue is present on the retreatment scan and how prior dose to that region should be counted. For example, a previous target region may later appear as aerated lung or scar. Counting it as normal lung may overestimate or underestimate risk, depending on whether it now contributes to lung function. These choices should be made for specific patients and organs, and they should be stated clearly.

Large changes such as atelectasis resolution or pleural effusion can make DIR especially uncertain. The algorithm may try to match collapsed to aerated lung, even though the change is not a simple deformation. In those cases, a deformably accumulated lung DVH may be less reliable than it appears. It may be better to review the prior and new dose distributions side by side, check the maximum or near-maximum dose to nearby serial organs, and test a few reasonable registration or overlap scenarios instead of relying on a single accumulated DVH.

Prior radiation can leave pneumonitis, fibrosis, scarring, volume loss, and local stiffness. These changes affect both anatomy and tissue tolerance. A cumulative dose map may look detailed, but it may still miss important differences in lung function. Ventilation, perfusion, or PET-based imaging may eventually help interpret cumulative lung dose, but these methods still need careful validation before routine use [43].

Central and ultra-central re-irradiation carry the greatest risk because critical structures may sit close to steep dose gradients, including the proximal bronchial tree, trachea, esophagus, heart, great vessels, pulmonary vessels, and spinal cord. In this setting, even a small registration error can matter. DIR-based accumulation should be checked carefully with visual review, landmark and contour assessment, dose-gradient review, and alternative registrations when needed. If uncertainty remains large enough to affect the plan, the accumulated DVH should be treated as an estimate rather than a precise value.

## 7. Adaptive Radiotherapy in the Re-Irradiation Setting

Adaptive radiotherapy changes cumulative dose assessment because the original plan and the dose actually delivered may no longer be the same. In conventional treatment, the baseline plan is often used as the best estimate of delivered dose. In adaptive workflows, daily anatomy may lead to plan-of-the-day selection, online adaptation, or replanning. When target size, atelectasis, lung inflation, or OAR position changes during treatment, the baseline plan may no longer reflect what the patient actually received [44,45].

In locally advanced NSCLC, adaptive radiotherapy has often been used to respond to tumor shrinkage, resolution of atelectasis, mediastinal change, and the need for better OAR sparing. In re-irradiation, these same issues matter even more because the margin for error is smaller. If an adaptive course is delivered near a previously irradiated esophagus, bronchial tree, heart, or spinal cord, day-to-day adaptation can change the cumulative dose to those organs. In some cases, it may also reduce OAR dose compared with a nonadaptive approach. For this reason, cumulative dose assessment should consider how well the baseline plan reflects the dose that was actually delivered [7].

A practical approach is often appropriate. If adaptation is mainly intended to maintain or reduce OAR exposure and daily anatomy remains fairly stable, as managed by the strategy of adapt-to-position in MR-guided ART, the baseline plan may still be a reasonable or conservative estimate [46]. If anatomy changes a lot, if OARs move in and out of steep dose gradients, or if a cumulative metric is close to a clinical limit, the adapted or delivered dose may need closer review. The report should clearly state whether cumulative dose was estimated from the baseline plan, a selected adapted plan, summed adapted fractions, or another approximation.

Adaptive workflows also increase the data-management burden. Multiple daily images, structure sets, dose files, and adapted plans may be involved, making standardized naming, verification, documentation, and peer review even more important. The basic clinical question remains simple: what dose distribution is being accumulated, and how well does it represent the dose the patient actually received?

## 8. Quality Assurance Framework

The central QA question is whether the cumulative dose result is reliable enough to guide the plan. Commissioning should therefore begin before registration and dose-accumulation tools are used routinely for lung re-irradiation. AAPM TG-132 provides general recommendations for image registration commissioning and QA in radiotherapy [24]. These tests are important because they use known or controlled cases, such as digital phantoms, physical or anthropomorphic phantoms, landmark-based datasets, 4D datasets, multimodality datasets, and selected clinical cases [27,28,29,47]. They help establish how the system behaves when the expected result is known or can be independently checked.

Registration accuracy and dose-accumulation validity should be evaluated separately. For practical QA, it is useful to consider six linked domains: (1) image-registration accuracy; (2) deformation-field behavior; (3) tissue correspondence when anatomy has disappeared, appeared, or changed composition; (4) dose-transfer accuracy, including interpolation, resampling, and energy/mass considerations; (5) biological-model uncertainty, including alpha/beta and recovery assumptions; and (6) clinical-decision sensitivity—whether reasonable variation in any of the preceding domains would alter the treatment decision. Confidence in one domain does not establish confidence in the others.

For lung re-irradiation, commissioning should reflect the clinical situations in which the tools will be used. Test cases should include peripheral and central targets, steep SBRT gradients, changing lung inflation, atelectasis, chest-wall proximity, mediastinal anatomy, and serial OARs. The goal is not only to confirm that images appear aligned overall, but to test whether the registration is accurate enough in the region where cumulative dose will be interpreted. Quantitative measures, such as the DICE similarity coefficient and Hausdorff distance for organ boundaries and the Jacobian determinant or inverse consistency error for internal deformation, may support this evaluation. However, good geometric agreement alone does not validate voxel-wise tissue correspondence or dose-transfer accuracy.

Dose-accumulation QA should also check the main steps that can affect the result, including coordinate handling, dose-grid resampling, interpolation, structure transfer, EQD2/BED conversion, respiratory-state handling, and reporting. High-gradient SBRT cases should be included because small spatial errors can create large changes in near-maximum dose. Conventional-to-SBRT and multiple-course scenarios should also be tested. If in-house scripts are used for biological conversion or cumulative metrics, they should be validated, documented, and version-controlled. When anatomy or density changes are large, energy- or mass-based checks may provide an additional review step [20,21,22,23].

Patient-specific QA remains necessary because real patients do not provide ground truth. A registration may pass commissioning tests and still be uncertain in a given re-irradiation case. Careful visual inspection remains the most feasible patient-specific evaluation in routine clinical practice and should be performed together with review of the accumulated dose distribution. Physician and physicist review should therefore focus on the anatomy and dose metrics that matter for the decision, not only on the global image overlay. The review should include the target region, OAR boundaries, bony anatomy, airways, vessels, chest wall, and relevant landmarks. Particular attention should be given to regions affected by surgery, tumor regression, atelectasis, fibrosis, or other major anatomic changes, as well as to areas receiving high dose or containing steep dose gradients. Propagated contours should be checked and corrected when needed. When available, DVF review should look for unrealistic expansion, compression, or folding. Alternative registrations should be compared when the result is close to a clinical limit or could change the plan.

The level of QA should match the clinical impact. The significance of a registration error depends not only on its spatial magnitude but also on its location: an error in a low-dose or low-gradient region may have little clinical effect, whereas a similar error near a steep gradient may substantially change the estimated cumulative dose. When practical, a suspected spatial mismatch should therefore be translated into an approximate dosimetric uncertainty. If cumulative dose is well below concern across reasonable methods, a simpler review may be sufficient. If a bronchial tree, esophageal, or spinal cord near-maximum dose is close to a limit, uncertainty becomes part of the decision. In those cases, comparison of rigid and deformable approaches, organ-specific registrations, conservative point-dose estimates, or scenario-based sensitivity checks may be needed. Where reproducibility itself is a concern, repeat evaluations are most informative when a relevant source of variability is deliberately changed—for example, observer, region of focus, initialization, registration strategy, or clinically reasonable parameter setting. Simply rerunning a deterministic algorithm under identical conditions does not meaningfully estimate uncertainty [48]. For point-dose estimates, the prior and current dose locations should be reviewed to ensure that the values correspond anatomically; directly summing spatially separated maximum doses may overestimate the dose delivered to any single location. The number and type of repeat evaluations should be driven by the software behavior, workflow, source of uncertainty being tested, and clinical consequence of the result.

AAPM TG-132 [24] provides useful general commissioning benchmarks for registration accuracy, but these should not be interpreted as organ-specific clinical safety thresholds. For example, a target-registration-error goal on the order of the maximum voxel dimension (commonly about 2–3 mm) and contour-surface agreement within expected contouring uncertainty have been used as QA benchmarks [24,47]. No validated evidence currently supports quantitative, distance-based registration metrics specific to the lung, esophagus, proximal bronchial tree, heart, great vessels, or spinal cord in re-irradiation. Clinical acceptability is instead organ-, location-, and dose-gradient dependent: the same 2–3 mm displacement may have little effect on a broad low-gradient lung metric but substantially change D0.03cc or D0.1cc for a serial OAR beside a steep SBRT gradient. A practical patient-specific criterion is therefore to test whether plausible registration perturbations materially change the relevant cumulative metric or the treatment decision. If they do, the accumulated value should be reported as uncertain, and a conservative alternative should be considered.

The accumulated dose should be assessed using clinically relevant metrics. Cumulative point-dose values should be compared with the corresponding locations and doses in the individual plans, while volumetric results should be reviewed using accumulated and individual DVHs, dose overlays, and relevant organ-specific metrics. Independent dose accumulation or quantitative registration analysis may be added when available, particularly when registration is uncertain, dose tolerance is approached, or the result is likely to alter the treatment decision. Documentation should make the work reproducible. The treatment record should state which prior courses were considered, what data were available, how registration and dose mapping were performed, what biological conversion and recovery assumptions were used, which cumulative metrics were reported, what major uncertainties remain, and the estimated upper and lower bounds of the cumulative dose. If another retreatment is considered later, the next team should be able to understand what was done and why [49,50,51]. Figure 3 highlights the relationship between registration uncertainty and dose error, and Table 4 lists patient-specific QA and reporting elements that should be documented.

## 9. Practical Clinical Considerations and Pathway

A practical approach to cumulative dose evaluation begins with the clinical question. The team needs to first define the retreatment scenario: treatment intent, target location, relevant OARs, expected overlap with prior dose, time since prior radiation, and the clinical risk of the decision. A small peripheral recurrence far from serial OARs does not require the same level of dose accumulation as an ultra-central recurrence beside the bronchial tree, esophagus, vessels, or spinal cord.

The next step is to verify the prior data. Prior images, structure sets, dose files, fractionation, respiratory management, and delivery records should be reviewed before any composite dose is trusted. If the prior data are incomplete, poor in quality, or not representative of what was delivered, the cumulative dose estimate should be kept conservative. If some structures are not available or not fully contoured, additional contouring may be necessary.

The third step is to judge the main uncertainty. Stable anatomy, similar respiratory state, and limited overlap may support a rigid review or conservative point-dose estimate. Moderate deformation, partial high-dose overlap, or different setup and breathing management may justify DIR accumulation. Major density or mass change, atelectasis resolution, pleural effusion, surgery, large tumor regression or progression, airway re-expansion, extensive fibrosis, incompatible respiratory states, or nonphysical deformation can weaken the link between registration and true tissue correspondence. In those cases, a single accumulated DVH may look more precise than it really is.

The dose-accumulation method should then be chosen to answer the clinical question with the least unnecessary complexity. If a conservative point-dose estimate resolves the OAR concern, full DIR accumulation may add little. If volumetric lung dose or complex high-dose overlap is central to the decision, three-dimensional accumulation may be useful, provided the registration is reliable. When fractionation differs and a biologically meaningful cumulative comparison is needed, doses should be converted to a common biological scale, such as EQD2 or BED, before they are added or compared. Physical-dose or qualitative overlap assessment may still answer selected spatial questions.

Finally, the result should be interpreted by risk level. In simple, low-risk cases, the simplest defensible method may be enough. In moderate-complexity cases, DIR can help when spatial information is reliable and affects the plan. In high-risk or high-uncertainty cases, especially central or ultra-central retreatment near serial OARs and steep gradients, cumulative dose should be treated as an estimate. Alternative registrations, conservative scenarios, or point-dose checks may be more useful than relying on one composite DVH. The final decision should also include expected benefit, prior toxicity, pulmonary function, comorbid lung disease, systemic therapy options, surgical or ablative alternatives, performance status, expected survival, and patient goals.

Figure 3 summarizes this practical pathway for selecting and interpreting cumulative dose methods in lung re-irradiation. Two hypothetical clinical workflow examples illustrating application of this pathway are provided in Appendix A and Appendix B.

## 10. Reporting Recommendations for Manuscripts and Clinical Reports

The re-irradiation literature remains difficult to interpret because cumulative dose methods are often underreported. The ESTRO-EORTC consensus called for standardized reporting in re-irradiation research, including prior and current treatment details, retreatment classification, target and OAR definitions, dose constraints, image registration, dose summation method, radiobiological considerations, toxicity reporting, and follow-up [6]. The ESTRO technical guidance and ReCOG consensus reports have extended that emphasis to incorporate technical methodology and uncertainty [7,8].

For lung re-irradiation studies, we suggest that a practical minimum reporting set include prior RT dose, fractionation, technique, target, dates, and delivered fractions; re-irradiation dose, fractionation, technique, target, breathing phase used for planning, and motion management; interval between courses; overlap classification; available DICOM data; registration method; dose mapping method; physical or biological dose, e.g., EQD2, or BED; alpha-beta values used for the dose conversion; tissue recovery assumptions; cumulative OAR constraints; structures revised or recontoured, metrics derived from 3D accumulation versus point-dose or DVH addition; uncertainty evaluation; toxicity grading system; follow-up schedule; and whether systemic therapy was concurrent or close in time.

Clinical reports can be shorter, but they should follow the same logic. They should document the method, assumptions, limitations, and potential clinical consequences. A strong report does not merely acknowledge that the composite plan was reviewed. It should nominally include details as discussed above, such as how dose was mapped and accumulated, which prior plans were included, uncertainty estimates and reliability of results, and what clinical decisions/actions were taken given the results.

## 11. Future Directions: From Technically Possible to Clinically Predictive

The next phase of lung re-irradiation research needs to move beyond technically possible dose accumulation toward clinically validated dose accumulation. Several areas deserve attention [52].

More clinically useful cumulative OAR constraints are needed, but they must remain linked to the methodology used to generate them. A bronchial-tree EQD2 maximum derived from deformable voxel warping is not automatically equivalent to one derived from point-dose summation. Published values should therefore be interpreted together with the endpoint, fractionation, biological model, recovery assumption, spatial accumulation method, and uncertainty; they should not be generalized as method-independent universal limits.

Future accumulation tools would be more clinically informative if uncertainty were reported explicitly. Instead of producing a single cumulative dose value, future systems should provide plausible ranges based on registration uncertainty, respiratory-state uncertainty, dose-gradient sensitivity, biological model/alpha-beta variation, and recovery assumptions. A cumulative spinal cord maximum dose estimate (D0.03cc) of 48 Gy EQD2 with low uncertainty is not clinically equivalent to 48 Gy EQD2 with high uncertainty. Furthermore, uncertainties interpreted with different error metrics are not necessarily consistent. Therefore, the clinical interpretation and application of dose estimates that reflect the underlying uncertainty would benefit from standardized interpretation, and future cumulative OAR constraints should ideally account for these uncertainty ranges.

Automated deformation QA is also needed. Jacobian maps, inverse-consistency metrics, landmark errors, contour mismatch, and dose-gradient sensitivity could be built into clinical workflows [53]. Artificial intelligence may help identify non-realistic registrations, but AI-assisted validation must be interpretable, transparent, and alternatively verifiable [54].

Biomechanical lung models may improve mapping across respiratory states and sliding interfaces. They may be particularly helpful when pleural motion and lung expansion dominate the uncertainty. Their use in routine practice will require commissioning standards and patient-specific validation.

Adaptive radiotherapy workflows will require practical estimation of delivered-dose accumulation. As online adaptive radiotherapy expands, the field will need efficient ways to store, identify, verify, and accumulate daily adapted dose. The baseline plan may remain a practical approximation in many situations, but high-risk re-irradiation near serial OARs will sometimes require a more detailed evaluation.

Proton therapy may offer dosimetric advantages in selected re-irradiation cases by reducing exit dose and limiting cumulative exposure to previously irradiated OARs [55]. Proton re-irradiation, however, introduces tissue heterogeneity, the need for careful characterization of tissue compositions, motion interplay, and other factors contributing to range uncertainties, as well as relative biological effectiveness assumptions. Dose accumulation must clearly distinguish physical dose from RBE-weighted dose and should account for changing lung density and anatomy [56].

PET-guided re-irradiation may improve patient selection, target delineation, and response assessment. FDG-PET can help distinguish recurrence from post-radiation fibrosis or atelectasis, define metabolically active sub-volumes, guide escalation or de-escalation, and potentially support dynamic dose management [57,58,59]. PET also introduces uncertainty from intrinsic reduced spatial resolution (relative to CT), respiratory motion, registration, segmentation, timing, and inflammatory uptake. PET should be used to support careful anatomic and dosimetric review, not replace it.

Delivered-dose verification is another area of future development relevant to cumulative dose assessment. In vivo and real-time dosimetry, including EPID-based systems, diodes, scintillators, and emerging detector platforms, can identify delivery deviations and may provide information closer to the dose actually delivered than the treatment plan alone [60]. Novel optoelectronic materials may eventually contribute to compact sensing technologies, but favorable photoresponse or photodetection properties do not by themselves establish suitability for megavoltage radiotherapy dosimetry. Radiation response, calibration stability, energy and dose-rate dependence, angular dependence, spatial and temporal resolution, and integration with patient geometry require dedicated validation. In re-irradiation, such measurements complement rather than replace image registration and dose mapping, because verified delivered dose still must be spatially related to anatomy across treatment courses.

Outcome-linked dose–response modeling is essential. The linear-quadratic model may not accurately predict the biological response of individual patients, particularly in previously irradiated tissues. For example, fibrotic tissue may tolerate additional radiation beyond that predicted by conventional radiobiological models. To improve clinical decision-making, prospective or high-quality retrospective datasets that include prior and current DICOM data, biological dose accumulation methods, uncertainty metrics, pulmonary function, systemic therapy, toxicity, local control, and other outcome measures, including quality of life, are highly encouraged. Only then can cumulative dose accumulation move from a planning exercise toward a predictive clinical tool.

## 12. Conclusions

Dose mapping and accumulation are central to lung cancer re-irradiation, but technical feasibility alone does not make them reliable. Re-irradiation in the thoracic area is met with several sources of uncertainty, including respiratory motion, sliding interfaces, tumor response, atelectasis, pleural effusion, fibrosis, density change, and steep dose gradients near critical organs. Rigid registration, DIR, direct dose interpolation, voxel warping, mass- and energy-based mapping, point-dose summation, and EQD2/BED conversion all have useful roles. Each method, however, depends on assumptions that need to be checked for the specific case.

The dose evaluation method should fit the clinical situation. In low-risk peripheral cases, rigid review or conservative point-dose estimates may be sufficient. In more complex cases with meaningful overlap, especially near central serial organs, DIR-based accumulation may add value when it is supported by patient-specific QA and careful uncertainty review. When major tissue replacement, density changes, or nonphysical deformation is present, cumulative dose maps need cautious interpretation and should not be used independently to drive clinical decisions.

For lung re-irradiation, the key question is whether the cumulative dose estimate is reliable enough to support the clinical action. A clear workflow, biological dose conversion, patient-specific QA, multidisciplinary review, and documentation of uncertainty provide the foundation for safer clinical use, more consistent dose reporting, and more reliable dose-volume-effect relationships in the re-irradiation setting. This Perspective therefore emphasizes matching the accumulation approach to the clinical question, the quality of the available data, and the level of uncertainty.

## Figures and Tables

**Figure 1:**
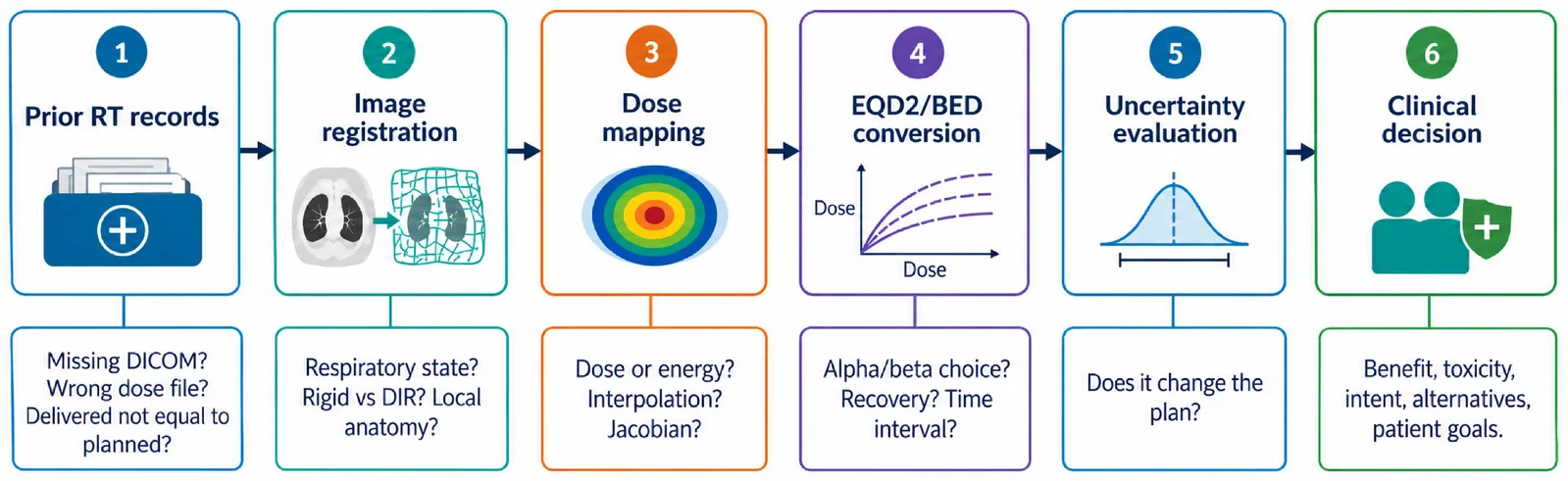
Key steps in cumulative dose evaluation for lung re-irradiation. The final DVH or dose metric depends on the quality of the prior records, the image registration, the dose-mapping method, the biological dose conversion, the uncertainty evaluation, and the clinical context in which the result is used.

**Figure 2:**
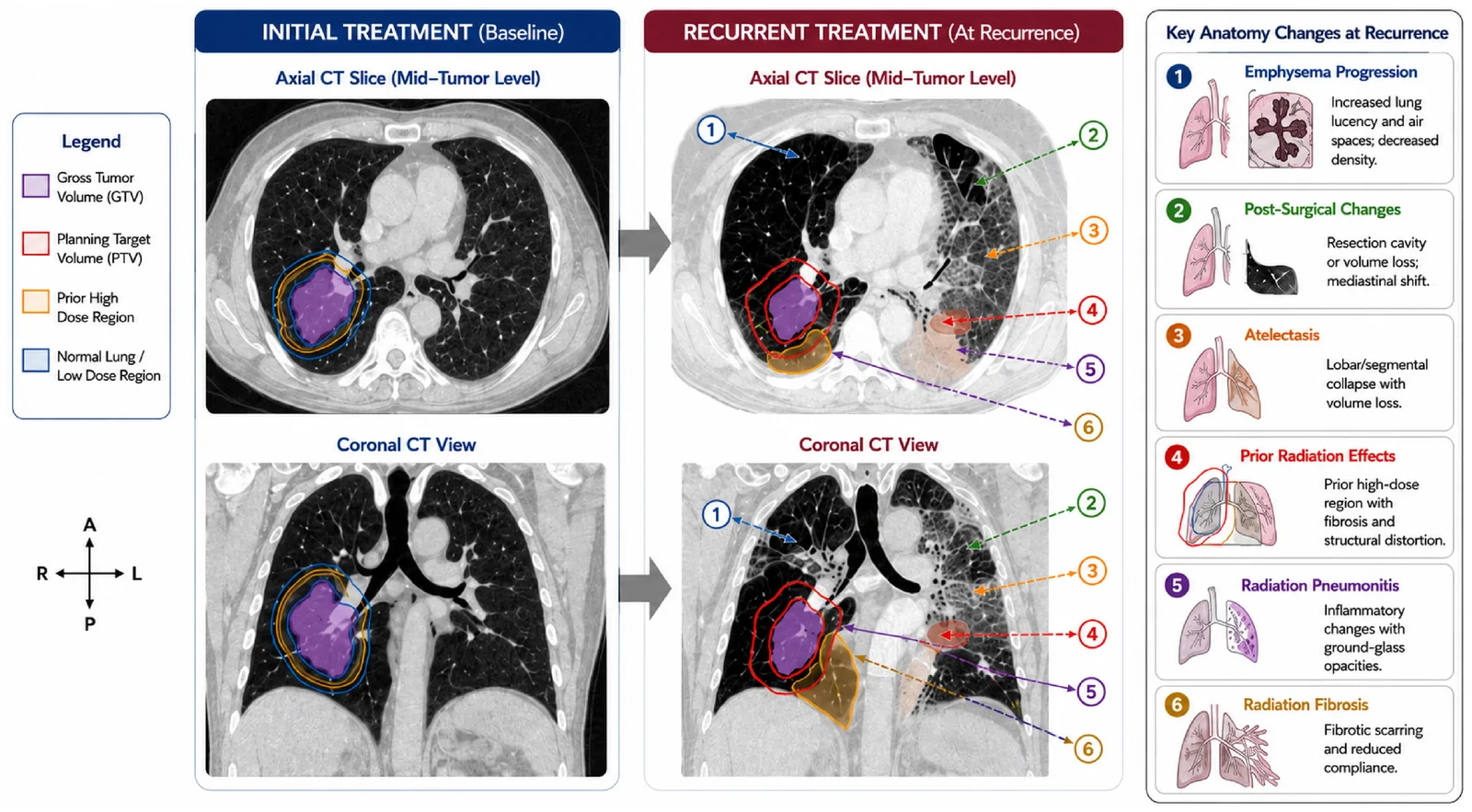
Synthetic conceptual illustration of potential tumor and thoracic anatomic changes between treatment courses, including emphysema, post-surgical change, atelectasis, prior radiation effects, pneumonitis, and fibrosis. Generated using OpenAI ChatGPT Plus platform, version GPT-5.6 Sol, 9 July 2026.

**Figure 3:**
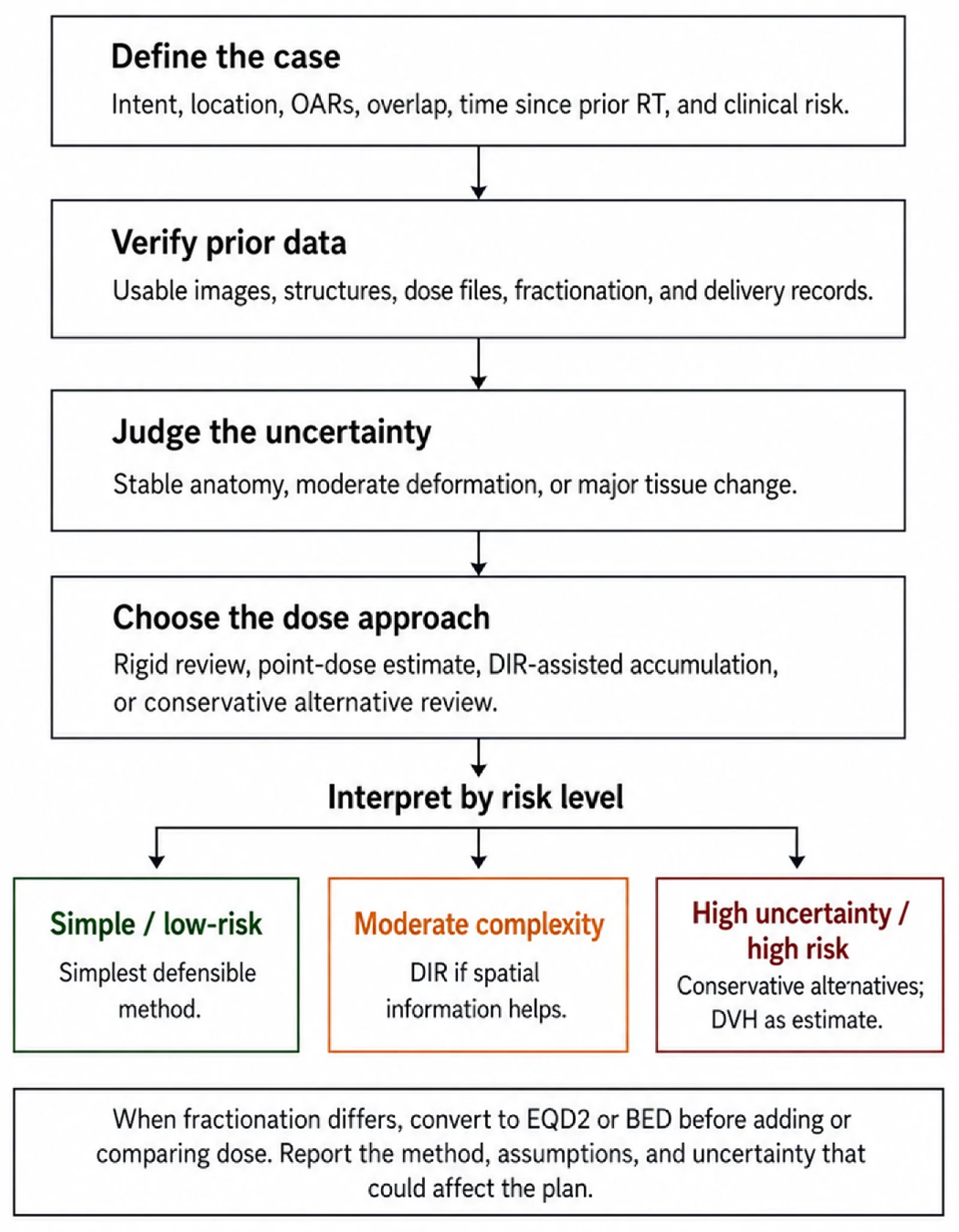
Practical pathway for selecting and interpreting cumulative dose methods in lung re-irradiation.

**Table 1:** Representative lung re-irradiation scenarios and the cumulative-dose question they raise.

Clinical Scenario	Preferred Initial Approach	Dominant Cumulative-Dose Question	Typical Structures of Concern
Peripheral recurrence after prior SBRT	Start with rigid or organ-specific rigid review; use DIR when anatomy has changed substantially or when the result may affect the plan.	Is there meaningful high-dose overlap with lung or chest wall?	Chest wall, ribs, lung-GTV, nearby small airways/vascular structures.
Central recurrence after definitive chemoradiation	Biologically corrected cumulative dose review, with physician/physicist evaluation and uncertainty checks.	Can serial OAR doses be kept within acceptable cumulative risk?	Proximal bronchial tree, trachea, esophagus, heart, vessels, spinal cord.
New primary in previously treated thorax	Classify the degree and location of overlap, identify the main organs at risk, and evaluate cumulative dose metrics that could affect the plan.	How much lung and heart are being re-exposed, and is there meaningful overlap with prior high-dose regions?	Composite doses for OARs, prior high-dose overlap region.
Oligometastatic thoracic retreatment	Use conservative point-dose review for serial organs when appropriate; use DIR when overlap is complex and may affect the plan.	Can re-irradiation be delivered without excessive cumulative dose to organs at risk near the prior field?	Depends on location; may include spinal cord, brachial plexus, esophagus, chest wall.
Palliative retreatment	Use simple conservative point-dose or field-overlap review for most palliative cases; consider a more detailed cumulative dose assessment when higher doses or longer expected survival make late toxicity more relevant.	What level of cumulative dose review is needed to avoid preventable severe toxicity while allowing timely symptom relief?	OARs adjacent to the symptomatic site; spinal cord and esophagus are often the main dose-limiting structures.

**Table 2:** DIR algorithm classes and practical tradeoffs for lung re-irradiation.

Registration/Algorithm Class	Core Assumption	Potential Strength in Lung	Major Lung-Specific Failure Mode
Global rigid registration	Thoracic anatomy can be approximated as one rigid body.	Transparent, reproducible, easily reviewed.	One match cannot simultaneously align tumor, spine, esophagus, lung, and chest wall when deformation is present.
Organ-specific rigid registration	Registration should focus on the organ at risk or region of interest most likely to influence the clinical decision.	Useful for serial OAR near-maximum estimates.	Different OARs may require different registrations; cumulative volumetric metrics become harder to interpret.
Intensity-based DIR	Similar image intensities represent corresponding tissue.	Can account for moderate anatomic changes between scans, including differences related to breathing.	Atelectasis, tumor response, inflammation, and effusion change intensity without true voxel correspondence.
B-spline/free-form DIR	Smooth deformation can represent anatomic change.	Flexible and widely implemented.	May smooth over pleural sliding or overfit if control grid is too fine or deformation scale is too large.
Demons/optical-flow-like DIR	Local intensity gradients can estimate local displacement.	Efficient and flexible.	May create nonphysical deformation near air-tissue interfaces or resolving collapse, and sensitive to imaging noise.
Contour- or feature-guided DIR	Clinically defined structures improve anatomic guidance.	Can guide the registration toward clinically important structures, such as the bronchial tree, esophagus, vessels, chest wall, or target region.	Contour agreement is not proof of voxel-level dose correspondence and reliable features do not always exist.
Biomechanical/hybrid DIR	Can guide the registration using tissue mechanics and selected landmarks or structures.	May help model breathing motion and pleural sliding.	Requires assumptions about tissue behavior, which may change after prior radiation because of inflammation, fibrosis, scarring, loss of elasticity or post- implantation of medical devices.

**Table 3:** Dose evaluation and dose mapping strategies in lung re-irradiation.

Dose Evaluation Strategy	Best Use	Strength	Key Limitation
Maximum or near-maximum point-dose summation	Serial OAR screening when data are incomplete or registration is unreliable.	Conservative approach that is easy to review, useful for spinal cord and other serial structures.	Separate maxima may arise at different locations; their sum is not a spatially accumulated maximum. Summed near-maximum metrics are not guaranteed upper bounds.
Metric addition	Cases where full dose grids are unavailable or registration is not reliable.	Course-specific mean doses may be added as organ-level summaries. For mean EQD2, each course’s dose distribution is converted before calculating the corresponding organ mean.	Other DVH metrics, such as D0.1cc, D1cc, or V20, should not be directly added because they may arise from different parts of the organ or represent different volumes in each course.
Dose overlay inspection	Qualitative review of field overlap or high-dose regions.	Simple visual communication tool.	Lack of quantitative or precise prediction. Physical dose summation is not biologically meaningful when fractionation differs.
Rigid 3D accumulation	Stable anatomy and limited deformation.	Reproducible and easy to QA.	Can be spatially inaccurate when local anatomy changes.
Voxel-based dose mapping/accumulation	Moderate deformation with good patient-specific QA.	Can provide spatial cumulative DVHs and high-dose overlap estimates.	Sensitive to DVF error, interpolation, non-invertibility, density change, and high gradients.
Energy/mass-congruent mapping	Regions with meaningful density or mass change.	Better reflects energy/mass conservation assumptions than direct dose interpolation in selected settings.	Not routinely available; still depends on tissue correspondence, image/density fidelity, transformation and implementation details, and does not solve tissue disappearance/replacement or biological uncertainty.
EQD2/BED biological accumulation	Different fractionation schedules.	Puts doses on a common biological scale before adding or comparing OAR dose.	Depends on chosen alpha-beta values and assumptions about tissue recovery and time between courses.

**Table 4:** Patient-specific QA and reporting checklist for cumulative dose evaluation.

QA/Reporting Item	Purpose	Minimum Documentation
Prior treatment verification	Avoid wrong-plan or planned-versus-delivered errors.	Dates, site, technique, prescription, delivered fractions, DICOM availability, delivery summary.
Contour review	Ensure relevant/overlap organs are contoured on both images, and consistent criteria are used for delineation.	Relevant organs added or modified contours for consistency (e.g., most superior and inferior slices of the heart), partially delineated contours.
Image registration review	Ensure spatial mapping is clinically meaningful.	Registration type, region of focus, OAR/target alignment, failed regions, reviewer names, or roles.
DVF plausibility	Identify nonphysical deformation.	Jacobian/folding review if available, landmark error, contour mismatch, sliding-interface concerns.
Biological conversion	Avoid invalid physical dose summation.	EQD2 or BED model, alpha-beta values, time between courses, recovery factors.
Cumulative metric selection	Choose metrics that fit the organ, clinical question, and confidence in the accumulation method.	Maximum or small-volume dose metrics for serial OARs; volumetric metrics only when spatial accumulation is reliable.
Uncertainty statement	Avoid false precision and estimate how uncertainty affects the clinical decision.	Main uncertainty sources; note whether reasonable alternative methods would change the conclusion.
Clinical action	Connect dosimetry to patient care.	Proceed, revise plan, alter prescription, add margin/constraint, choose alternate therapy, or defer.
Tissue-correspondence validity	Identify regions in which an anatomic match may not represent the same tissue.	Document tissue loss/gain, re-aeration, collapse, cavitation, surgery, fluid change, or other regions where one-to-one correspondence is questionable.
Dose-transfer validity	Separate geometric alignment from dosimetric correctness.	Document interpolation/resampling approach, grid resolution, coordinate handling, and any energy/mass-aware method or independent check used.
Biological-model uncertainty	Make EQD2/BED and recovery assumptions explicit.	Document alpha/beta values, fractionation, model limitations at high dose per fraction, recovery assumption, and sensitivity analysis when clinically relevant.
Clinical-decision sensitivity	Determine whether uncertainty is large enough to impact clinical decision-making.	State whether reasonable alternative registrations, dose-transfer methods, or biological assumptions would change the plan or clinical recommendation.

## Data Availability

No new data were created or analyzed in this study. Data sharing is not applicable to this article.

## Appendix A

**Illustrative Clinical Workflow Example 1:**

**Peripheral Re-Irradiation with Relatively Stable Anatomy**

The following hypothetical example illustrates the application of the cumulative-dose workflow in a relatively straightforward re-irradiation setting.

**Hypothetical scenario.** A patient previously received SBRT for a peripheral right-upper-lobe NSCLC and returns several years later with a new peripheral lesion adjacent to, but not centered within, the prior high-dose region. Prior treatment images, structures, dose information, and delivery records are available. The current CT planning images show similar lung inflation and stable rib and chest-wall anatomy, without major atelectasis, pleural effusion, or postoperative distortion. The clinical question is whether overlap between the prior and prospective high-dose regions meaningfully affects lung or chest-wall dose.

**Workflow.** The prior and current treatment information is reviewed to confirm the treatment history, respiratory state, and anatomy relevant to the region of interest. A local rigid registration focused on the ribs, chest wall, and adjacent lung is performed and visually reviewed using bony anatomy and the target region. Because the anatomy is relatively stable, rigid registration is used as the primary approach, while DIR may be used as a sensitivity check rather than assumed to provide a more reliable result. When fractionation differs between courses, doses are converted to a common biological scale before clinically relevant lung and chest-wall metrics are compared [7].

**Illustrative result.** Suppose the clinically relevant lung and chest-wall dose estimates remain below the level of concern across the reasonable registration approaches, and the location and extent of the high-dose overlap remain essentially unchanged. In this setting, the small variation between approaches would not alter the clinical interpretation or treatment plan.

**Interpretation.** The cumulative-dose estimate can therefore be considered sufficiently reliable for the clinical decision, while the registration method, biological-conversion assumptions, and sensitivity assessment are documented. This example illustrates a setting in which a relatively simple and transparent accumulation approach may be sufficient because reasonable methodological variation does not change the clinical conclusion.

## Appendix B

**Illustrative Clinical Workflow Example 2:**

**Central Re-Irradiation in Which Uncertainty Changes the Plan**

The following hypothetical example illustrates application of the cumulative-dose workflow in a more complex setting in which uncertainty may affect the clinical decision.

**Hypothetical scenario.** A patient previously received conventionally fractionated chemoradiation for a central NSCLC and returns several years later with a central/hilar recurrence. The original course delivered 60 Gy in 30 fractions, and the proposed re-irradiation course is hypofractionated. The new target lies close to the proximal bronchial tree, esophagus, and spinal cord. Between treatment courses, prior atelectasis has partially resolved and the mediastinal and airway anatomy has changed. The clinical question is whether cumulative dose to the proximal bronchial tree can be estimated reliably enough to support the proposed retreatment.

**Workflow.** The prior and current treatment information is reviewed to confirm the treatment history, respiratory state, and anatomy relevant to the organs at risk. A spine-focused rigid registration is reviewed for the spinal cord, while a separate local registration centered on the central airways and mediastinum is evaluated for the proximal bronchial tree and esophagus. DIR is used only where tissue correspondence appears sufficiently reliable; the region affected by resolved atelectasis is treated as uncertain rather than assumed to have one-to-one voxel correspondence. When fractionation differs between courses, doses are converted to a common biological scale, and cumulative near-maximum doses are compared across several clinically reasonable registration scenarios [7].

**Illustrative result.** Suppose these reasonable approaches produce a cumulative proximal-bronchial-tree Dmax range of approximately 106–113 Gy EQD2, spanning the approximately 110 Gy composite reference summarized from the 2025 American Radium Society guidance [5]. In this setting, reporting a single value such as 109 Gy would imply greater precision than the analysis supports. Because the range crosses a clinically relevant reference value, the uncertainty could affect the treatment decision.

**Interpretation.** The team may therefore revise the retreatment plan to reduce dose to the proximal bronchial tree, use a conservative estimate when evaluating cumulative risk, and document the plausible dose range rather than a single composite value. This example illustrates a setting in which methodological uncertainty is clinically important because reasonable variation in registration and dose accumulation changes the interpretation and may alter the treatment plan.
